# Clonality Analysis for the Relationship between the Pulmonary Combined Neuroendocrine Carcinoma and “the So-Called Reported Histologic Transformation”

**DOI:** 10.3390/cancers15235649

**Published:** 2023-11-29

**Authors:** Haiyue Wang, Yanli Zhu, Wei Sun, Xin Yang, Xinying Liu, Kaiwen Chi, Xiaozheng Huang, Lixin Zhou, Weijing Cai, Dongmei Lin

**Affiliations:** 1 Key Laboratory of Carcinogenesis and Translational Research (Ministry of Education), Department of Pathology, Peking University Cancer Hospital & Institute, Beijing 100142, China; 1711110472@bjmu.edu.cn (H.W.); 2111120014@bjmu.edu.cn (Y.Z.); swsu8796@163.com (W.S.); emily106_2@163.com (X.Y.); xinying_liu@bjmu.edu.cn (X.L.); chikaiwen@bjmu.edu.cn (K.C.); hxz182182@163.com (X.H.); zlixin@aliyun.com (L.Z.); 2 Shanghai Tongshu Biotechnology Co., Ltd., Shanghai 200120, China

**Keywords:** combined neuroendocrine carcinoma, histologic transformation, whole exome sequencing, clonality analysis, immunohistochemistry, RB1

## Abstract

**Simple Summary:**

Histologic transformation has been increasingly common in clinical. However, whether the transformed tumor to be a new component or a combined tumor remains controversial. This study aimed to explore the relationship and focused on 21 combined neuroendocrine carcinoma. The frequency of p53 inactivation as assessed by immunohistochemistry in NEC and non-NEC components was 76.2 and 76.2%, and for Rb, it was 66.7 and 61.9%, respectively. For molecular alterations assessed by whole exome sequencing, the frequency of mutations in *TP53*, *RB1*, and *EGFR* in NEC and non-NEC components was 81.0/81.0%, 28.6/28.6%, and 42.9/42.9%, respectively. Immunohistochemistry was relatively more sensitive in Rb detection. The different components were found to have a common clonal origin based on the clonal evolution analysis of all 21 cases, which was consistent with previous studies on “HT”. Our findings highlight the significance of evaluating the protein expression and gene status of TP53, RB1, and EGFR to discover the potential combined component or recognize the potential transformation cases. Therefore, our findings have strong implications in the clinical assessment of combined tumors.

**Abstract:**

Histologic transformation (HT) is common following targeted therapy in adenocarcinoma. However, whether the transformed tumor is a new component or a combined neuroendocrine carcinoma (C-NEC) remains controversial. We aimed to explore the relationship between pulmonary C-NEC and HT. Macro-dissection was performed on different components of surgically resected C-NEC samples. Molecular alterations and clonal evolution were analyzed using whole exome sequencing (WES). The gene statuses for *TP53* and *RB1* were determined using immunohistochemistry (IHC) and WES to analyze the relationship between C-NEC and reported HT. Sixteen combined small-cell lung cancer patients and five combined large-cell neuroendocrine carcinoma patients were enrolled. The frequency of p53 and Rb inactivation, assessed using IHC in NEC and non-NEC components, was 76.2/76.2% and 66.7/61.9%, respectively. The expression consistency between the components was 81.0 and 85.7% for p53 and Rb, respectively. The frequencies of *TP53*, *RB1*, and *EGFR* mutations, assessed using WES in NEC and non-NEC components, were 81.0/81.0%, 28.6/28.6%, and 42.9/42.9%, respectively. The concordance rates for *TP53*, *RB1*, and *EGFR* were 90.5, 71.4, and 90.5%, respectively. The consistency rate between IHC and WES was 81.0 and 61.9% for *TP53* and *RB1*, respectively. The different components had a common clonal origin for the 21 C-NECs in the clonal analysis, consistent with previous studies on HT. Our study shows that IHC is more sensitive for Rb detection and C-NEC, and the reported HT may be due to differences in evaluations between pathologist and clinicians. Assessing the p53/Rb and *EGFR* status for such cases would help in recognizing potential transformation cases or uncovering potential combined components.

## 1. Introduction

Combined small-cell lung cancer (C-SCLC) is a combination of pure SCLC and non-small cell lung cancer (NSCLC). It is relatively rare, accounting for 2–28% of all SCLC cases [1,2]. Large-cell neuroendocrine carcinoma (LCNEC) is another neuroendocrine carcinoma (NEC), diagnosed in approximately 3% of all patients with lung cancer. Similarly, it is common for LCNEC to occur in combination with other lung cancer types, such as SCLC, adenocarcinoma (ADC), and squamous cell carcinoma (SCC). According to the latest report of the World Health Organization (WHO) regarding thoracic tumors, combined LCNEC (C-LCNEC) accounts for approximately 20% of resected LCNEC cases [3].

In SCLC, *TP53* and retinoblastoma susceptibility gene (*RB1*) inactivation are the most common molecular alterations, of which *TP53* and *RB1* mutation rates range between 45–90% and 23.8–67% [4,5,6], respectively, suggesting that *TP53* and *RB1* mutations play an important role in the carcinogenesis of SCLC. However, given the few cases and strict diagnostic criteria, studies on molecular alterations related to LCNEC are limited. Tomohiro Miyoshi et al. [7] sequenced the coding exons of 78 LCNECs and showed that the *TP53* and *RB1* inactivation rates were 71% and 26%, respectively. Other studies reported that the frequency of *RB1* mutations in LCNEC ranged between 30 and 35% [8,9], which is lower than that in SCLC. The occurrence of epidermal growth factor receptor gene (*EGFR*) mutations in SCLC [5,10], LCNEC, or SCC is extremely rare; much lower than that in ADC [11]. However, while most studies have focused on pure NECs, C-NECs must be screened to determine the frequency of mutations in these genes.

Among the detection methodologies, the advantages of whole exome sequencing (WES) technology are manifold, including improved sensitivity in gene mutation detection, fast turnaround time, and reduced costs compared to traditional sequencing methods. Thus, WES has been widely used and applied to characterize the genetic profiles of patients. However, only a few studies have used immunohistochemistry (IHC) instead of WES [12] to determine the status of the *RB1*. Some studies have reported that IHC is a reliable alternative assay for *RB1* mutation analysis [13,14]. In contrast, some studies have used only one detection method. Therefore, the concordance rate between the two detection methodologies must be analyzed, particularly for *RB1*.

The phrase “SCLC transformation” is increasingly being used in clinical literature, especially for patients diagnosed with EGFR mutation-positive NSCLC after receiving EGFR tyrosine kinase inhibitor (TKI) therapy [15,16]. However, SCLC transformation can also occur in anaplastic lymphoma kinase (ALK)-positive lung cancer after treatment with ALK inhibitors [17] and in wild-type EGFR or ALK NSCLC treated with immunotherapy [18]. Except for SCLC, the histologic subtypes after transformation also contain LCNECs [19] and SCCs [20] as the rare transformed histological phenotypes. However, most reported transformed cases were initially diagnosed as ADC, as assessed using tissue biopsy, fine-needle aspirates, or pleural fluid cytology assays after the disease progression that were re-biopsied as SCLC [15,18,21]. Hence, it is possible that the biopsy or cytologic samples used to make the initial and post-transformed diagnoses do not provide sufficient pathological material to reveal the presence of combined histology. Therefore, for histologic transformation (HT), two views are prominent at present. One is that non-NEC and NEC components have a common cell origin [13,22] and are transformed into SCLC under the pressure of TKI treatment [23], and the other is that they are initially C-NEC [24]. Due to recent improvements in diagnosis and treatment technology and early intervention, the so-called HT has been much more commonly encountered following the treatment of these tumors than imagined. Therefore, deep consideration is warranted to evaluate the phenomenon of transformation.

Herein, we collected tissue samples of combined carcinoma, analyzed their different components using molecular and clone analysis, and discussed the internal relationship with the transformation cases reported in previous studies. In addition, we studied the clinicopathological and immunohistochemical features and other common gene alterations in C-NEC.

## 2. Materials and Methods

### 2.1. Patient Cohort

A total of 189 NEC cases in patients who underwent a pulmonary lobectomy or wedge resection from 2011 to 2021 at Peking cancer hospital (Peking, China) were retrieved from the pathology database. Of these, cases with more than 50% tumor content without extensive necrosis, which were efficiently separable, were screened using molecular detection and enrolled in our study. Finally, 20 C-NEC cases were included, constituting the experimental group. One case, which was collected in 2021 and was finally diagnosed as C-SCLC based on the metastasis to the liver and diaphragm, was regarded as the validation group. The diagnosis underwent a blinded review process, in which three experienced pathologists (Dongmei Lin, Haiyue Wang, and Yanli Zhu) independently reviewed the slides without their clinical details. The diagnosis and histopathological characteristics were confirmed based on the fifth edition of the WHO criteria for lung neuroendocrine tumors. The staging was undertaken following the eighth edition of the Cancer Staging Manual released by the American Joint Committee on Cancer for tumor, lymph node, and metastasis classification. The clinicopathological parameters were obtained from medical records and telephone interviews. Informed consent was obtained from all the patients.

### 2.2. Immunohistochemistry for Determining the Expression of p53 and Rb

Serial sections with a thickness of 4 μm from the whole formalin-fixed paraffin-embedded samples of C-NECs were sliced and placed onto glass slides, followed by the IHC assay. Evaluation of p53 expression was performed using a mouse anti-p53 monoclonal antibody (clone DO-7; Gene Tech, Shanghai, China) at a working concentration. The expression of p53 was categorized as complete absence, overexpression, and wild-type. Among these, complete loss of expression or overexpression in ≥70–80% of tumor cells were interpreted as p53 inactivation [25]. Slides stained with Rb were labeled using a mouse anti-Rb monoclonal antibody (clone 13A10; ZSGB-BIO, Beijing, China) at a working concentration. The Rb IHC results were classified as mutant type (complete absence, no nuclear expression) and wild type [13].

### 2.3. Tissue Dissection for C-NEC

The enrolled specimens stained with hematoxylin and eosin (HE) were reviewed to determine their tumor contents. Areas purely composed of NEC or NSCLC were delineated carefully in each case. The HE-stained sections were compared, followed by accurately marking the different tumor areas in the unstained sections. Unstained tumor tissue sections with different components were then macro-dissected manually. Additional HE staining was performed on the cut sections to ensure dissection accuracy. A neoplastic cellularity of at least 50% was obtained for all the tumor samples.

### 2.4. Whole Exome Sequencing for C-NEC

The DNA was extracted from formalin-fixed paraffin-embedded tissue samples using a Tissue Kit (69504; QIAGEN, Venlo, The Netherlands) following the manufacturer’s instructions. The DNA was isolated using targeted capture pulldown. Exon-wide libraries were generated from the native genomic DNA using the xGen^®^ Exome Research Panel (Integrated DNA Technologies, Skokie, IL, USA) and a TruePrep DNA Library Prep Kit V2 for Illumina (#TD501; Vazyme, Nanjing, China) following the manufacturer’s instructions. Paired-end sequence data were generated using a NovaSeq 6000 machine with an average sequencing depth of 185× for adjacent normal tissue as a control and 245× for the tumor tissues. The sequencing data were aligned to the human reference genome (NCBI build 37) using the Burrows–Wheeler Aligner. Polymerase chain reaction duplicates were sorted and removed using Sambamba, and the base quality score was recalibrated using GATK 4.1.

### 2.5. Data Processing for C-NEC

Single nucleotide variants (SNVs), insertions, and deletions were detected using Strelka2. Variants and polymorphisms were annotated using Annovar. A minimum of 20 reads covering the mutated region and 5 reads supporting the variant allele are required for somatic SNV/ indel calling. In contrast, the sequencing depth needs to be ≥20×, and the reads supporting the variant must be <5 at the same site in the normal control sample. We used the Strelka2 software to detect somatic mutations by analyzing paired tumor samples and matched control samples. During the detection, the algorithm automatically removed germline mutations. Variants were annotated using the ExAC, gnomAD, and esp6500 databases, and those with a MAF >1% were labeled as benign variants. Somatic copy number variations (CNVs) were analyzed using FACETS, and the resulting CNVs were used in further analysis.

### 2.6. Clonal Analysis for C-NEC

PyClone (version 0.13.1) was used to cluster subclones inferred from SNVs. Optimal tree solutions were obtained using the iterative version of CITUP, with the clustering results used as the input. A fish plot and phylogenetic tree of each patient were constructed using timescape, and a two-dimensional plot reflecting the cancer cell fraction (CCF) of each mutation was plotted using the R package ggpubr. Based on the clonal evolution theory of tumorigenesis, tumors arising from a single ancestral progenitor cell are assumed to inherit identical somatic variants. Thus, tumor pairs were defined as having a common clonal origin when the clonal relationship between paired tumor components met the following criteria: (i) possibly damaging clonal driver mutations were shared by two tumor components, (ii) possibly damaging driver mutations were clonal in one tumor component but subclonal in another paired tumor component, (iii) ≥2 clusters with passenger mutations were shared by two tumor components and were clonal in at least one tumor component. Our driver gene set was built by combining two driver gene lists defined in previous studies [26,27]. The mutations in driver genes were defined as possibly damaging as follows: (i) nonsense, frame-shift, and splice-site mutations; and (ii) missense mutations either with a FATHMM-MKL score >0.5 in the annotation of the Catalogue of Somatic Mutations in Cancer, or identified to be functionally damaging by two or more functional analysis algorithms, i.e., predication score of 0.0–0.05 using SIFT, “possibly damaging” or “probably damaging” using Polyphen2, “medium” or “high” using Mutation Assessor, or a predication score of >0.5 using FATHMM-MKL.

### 2.7. Statistical Analyses

The characteristics of the clinicopathological data were assessed using SPSS software, version 23.0 (SPSS, Chicago, IL, USA), and the *χ^2^* test was used for the analysis. Statistical significance was set to a *p*-value < 0.05.

## 3. Results

### 3.1. Clinicopathological Characteristics of the Patients

**Figure 1:**
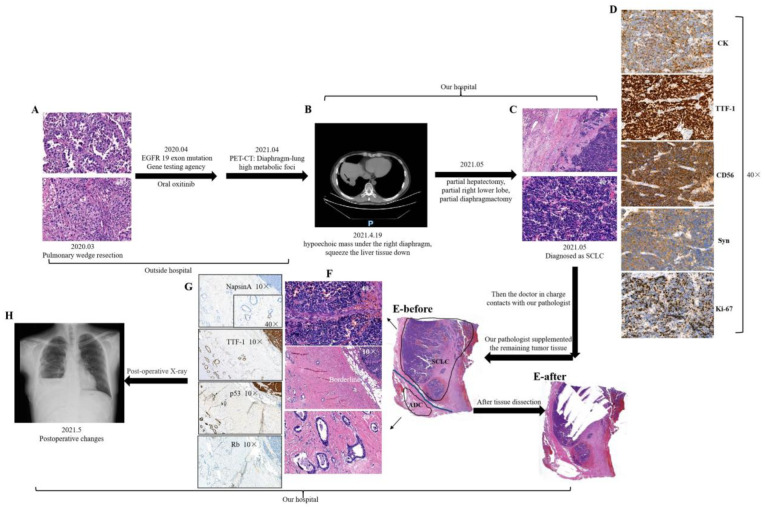
Case T21, which was initially diagnosed as ADC in an outside organization, predominantly had an acinar growth pattern with a partially with solid structure (**A**). After taking oxitinib orally for one year, the patient’s PET-CT and chest CT showed a hypermetabolic focus and abnormal mass under the right diaphragm and invasion to the diaphragm, right lung parenchyma, and liver tissue (**B**). Then, the patient received a partial hepatectomy with a partial right lower lobe and partial diaphragmactomy. Postoperative pathological HE images are shows in (**C**), which were diagnosed as SCLC. The immunohistochemistry result is exhibited in (**D)**, with CK, TTF-1, CD56, and Syn positive for SCLC. The Ki-67 index was approximately 70%. Due to the inconsistent diagnosis, the pathologist supplemented the remaining tumor tissue. Only a small-sized ADC component was observed in the diaphragm tissue (**E**) before, (**F**)). Similarly, the tissue after manual dissection was stained with HE (**E**) after. Also, the ADC component was confirmed by NapsinA (exhibited in a higher magnification in the lower right (40×) and TTF-1 (**G**)). p53 was 95% diffuse and strongly positive, and Rb was negative in both components. The X-ray images showed postoperative changes after resection of the lung and diaphragm (**H**).

The clinicopathological characteristics of 21 patients (16 C-SCLC and 5 C-LCNEC) diagnosed with C-NEC are described in detail in Table 1 and Table S1. The proportion of the SCLC components ranged between 5 and 98%, and that of LCNEC was between 45 and 95%. Of the 16 C-SCLC cases, 15 were combined tumors with ADC and 1 was with SCC. One C-SCLC case, initially diagnosed as ADC and then as SCLC, had received EGFR-targeted therapy and was regarded as an “SCLC transformation”. However, it later proved to be a combined SCLC/ADC after sufficient sampling (Figure 1). Of the five C-LCNEC cases, three were combined tumors with ADC and two were with SCC. These cases included 18 males (13 in the C-SCLC and 5 in the C-LCNEC cohort) and 3 females (all in C-SCLC). The median age was 62 years (range: 35–83 y). The median mitotic figure in the NE and non-NE components were 31 and 2 per mm^2^, respectively. The median tumor size was 3.4 cm. Notably, 10 tumors were of the peripheral type and 11 were of the central type. Six patients were subjected to neoadjuvant chemotherapy before surgery. Ten patients were never smokers, whereas eleven were currently smoking (1/21, 4.8%) or ex-smokers (10/21, 47.6%). Five cases presented a family history of cancer (hepatocellular carcinoma, gastric ADC, or lung cancer). Lymph node metastasis was detected in six patients. The tumors were classified as stage I (n = 11), stage II (n = 6), stage III (n = 3), and stage IV (n = 1).

### 3.2. Expression of p53 and Rb Assessed Using IHC

The two components of the combined tumors were analyzed separately in each case, and protein inactivation in any component was determined to be a mutation. Inactivation of p53 was found in both components of 14 cases, of which 9 showed diffuse but strong expression and 5 exhibited a complete loss of expression. Three of the remaining seven cases exhibited a wild-type expression pattern with different degrees of positive expression. Inactivation of p53 was found in the NEC, and wild-type expression patterns were found in the non-NEC components of two other cases. The other two cases showed a reverse trend with inactivation in the non-NEC and wild-type expression in the NEC components. In summary, 18 cases presented with p53 inactivation, assessed using the IHC assay, and the frequency of p53 inactivation in both the NEC and non-NEC components was identical, i.e., 76.2% (16/21). Identical expression of p53 in the two tumor components was found in 17 cases (81.0%, 17/21; 14 with inactivation, 3 with wild-type expression) (Table 2).

From the cohort, 12 cases exhibited a loss of expression of Rb within both components, whereas 6 cases showed a wild-type expression pattern. The other two cases presented a loss of expression in the NEC components, whereas the non-NEC components showed wild-type Rb expression. The remaining case had wild-type Rb expression in the NEC and a loss of expression in the non-NEC component. In summary, 15 cases presented with a loss of expression of Rb in the IHC assay, with a frequency of 66.7% (14/21) and 61.9% (13/21) in the NEC and non-NEC components, respectively. Identical expression of Rb in the two components was found in 18 cases (85.7%, 18/21; 12 with inactivation and 6 with wild-type expression) (Table 2).

Taken together, in the IHC assay, a total of nine cases (42.9%, 9/21) presented with simultaneous p53/Rb inactivation in the two different tumor components, two cases presented with a simultaneous wild-type expression pattern of p53/Rb, and the remaining ten cases presented with inconsistent expression of p53 and Rb.

### 3.3. Molecular Alterations

#### 3.3.1. Mutations in TP53 and RB1

**Figure 2:**
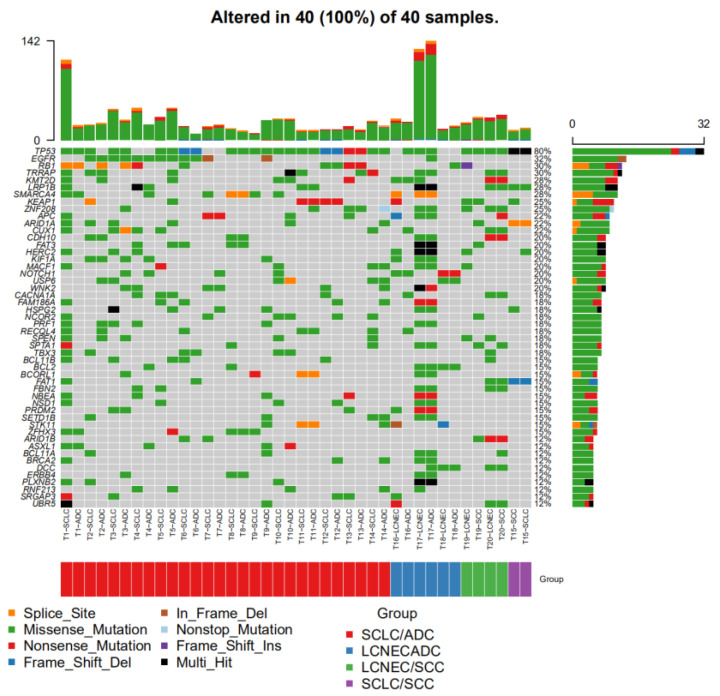
Common driver gene analysis in 20 C-NEC (except case T21). The C-NEC was classified as SCLC/ADC, SCLC/SCC, LCNEC/ADC, LCNEC/SCC, forming four groups. *TP53*, *EGFR,* and *RB1* were the most common molecular alterations.

To explain the protein expression patterns, we performed WES. Of the 21 C-NEC cases (Figure 2, Supplementary Figure S1A), 16 (76.2%) had *TP53* mutations and 3 (14.3%) contained the wild-type *TP53* gene within both components. Another two cases (T2, T16) showed inconsistent *TP53* mutation in the two components; one with NEC+/non-NEC−, the other with NEC−/non-NEC+. Therefore, the total proportion of cases of different components simultaneously with and without *TP53* mutation was 90.5% (19/21) (Table 3). In addition, the frequency of *TP53* mutations in the NEC and non-NEC components was 81.0% (17/21) and 81.0% (17/21), respectively. Considering the overall p53 IHC results, the concordance rate between *TP53* and p53 IHC was 81.0% (17/21) (Table 4).

For *RB1*, only 3 cases exhibited consistent molecular alterations, and 12 cases contained the wild-type *RB1* gene in both components. Inconsistent results were obtained between the NEC and non-NEC components in six cases (three with mutations exclusively in the NEC component and three with mutations exclusively in the non-NEC component). Therefore, the total proportion of cases of different components simultaneously with and without *RB1* mutation was 71.4% (15/21) (Table 3). The frequency of *RB1* mutations in the NEC and non-NEC components was 28.6% (6/21) and 28.6% (6/21), respectively. Upon comparison of the consistency of alteration in protein expression (IHC) and gene expression (WES) in each individual, a consistency rate of 61.9% (13/21) was obtained (Table 4). The detailed data are presented in Table S1.

Taken together, the results of the WES indicate a total of 3 cases (14.3%, 3/21) with simultaneous *TP53* and *RB1* mutation, 1 with simultaneous *TP53* and *RB1* wild-type sequence, and the remaining 17 cases with inconsistent alterations in *TP53* and *RB1* (Table 3).

#### 3.3.2. *EGFR* Mutation

According to the driver gene analysis of the findings of the WES performed for the 21 C-NEC cases, *TP53* (85.7%, 18/21), *RB1* (42.9%, 9/21), and *EGFR* (42.9%, 9/21) were the most common molecular alterations (Figure 2, Supplementary Figure S1A). In 16 C-SCLCs, 50% (8/16) of the cases had *EGFR* mutations. The different components simultaneously contained *EGFR* mutations, accounting for 31.3% (5/16). In five C-LCNECs, only one ADC component contained an *EGFR* mutation (p.D1168H) (Table 4, Table S1). In addition, in the 21 C-HGNEC, 10 cases underwent preoperative biopsy or surgery resection, and 6 cases were diagnosed with ADC or SCC. However, of the six cases, only case T21 (Supplementary Figure S1D) underwent WES analysis, and the remaining failed due to an insufficient sample size or unqualified quality control.

Based on our laboratory ARMS PCR data (Table S1) and WES analysis, we can conclude that the percentages of *EGFR* mutations in the non-NEC (all ADC) and NEC (all SCLC) components were 42.9% (9/21), 42.9% (9/21), respectively. No *EGFR* mutations were found in SCC or LCNEC. The percentages of the simultaneous presence and absence of the *EGFR* mutations in both components among the cohort were 38.1% (8/21) and 52.4% (11/21), respectively. Inconsistent *EGFR* mutations in the two different components were found in two cases (9.5%; T7 and T17), which exclusively occurred in the SCLC (*EGFR p.E746_A750del*) component and the ADC (*EGFR p.D1168H*) component, respectively.

#### 3.3.3. Molecular Alterations in Other Common Genes

A search for a common driver gene in the SCLC/SCC group yielded only the *TP53* mutation (Supplementary Figure S1). In the LCNEC/ADC group, *TP53* (66.7%), *BCL2* (66.7%), *NOTCH1* (66.7%), *APC* (66.7%), *KMT2D* (66.7%), and *POLE* (66.7%) exhibited the highest frequencies of mutated genes (Supplementary Figure S1). Similarly, no significant difference was observed in the variation frequency between the ADC and LCNEC components. In the LCNEC/SCC group, *TP53* mutations were detected in both components in two cases, and no *EGFR* mutations were observed.

Two cases of LCNEC/ADC (T16, T18) were identified with identical *NOTCH1* mutations in both components, i.e., *NOTCH1 p.A1471V* and NOTCH1 p.Q1247*, respectively. The SCLC component of SCLC/ADC (T10) showed a *NOTCH1 (NOTCH1 p.G1034D)* mutation. The ADC component of SCLC/ADC (T3, T7) showed a *NOTCH1* mutation, with *NOTCH1 p.S2136L* and *NOTCH1 p.R234C*, respectively (Table S1).

### 3.4. Clonal Evolution Analysis for C-NEC

**Figure 3:**
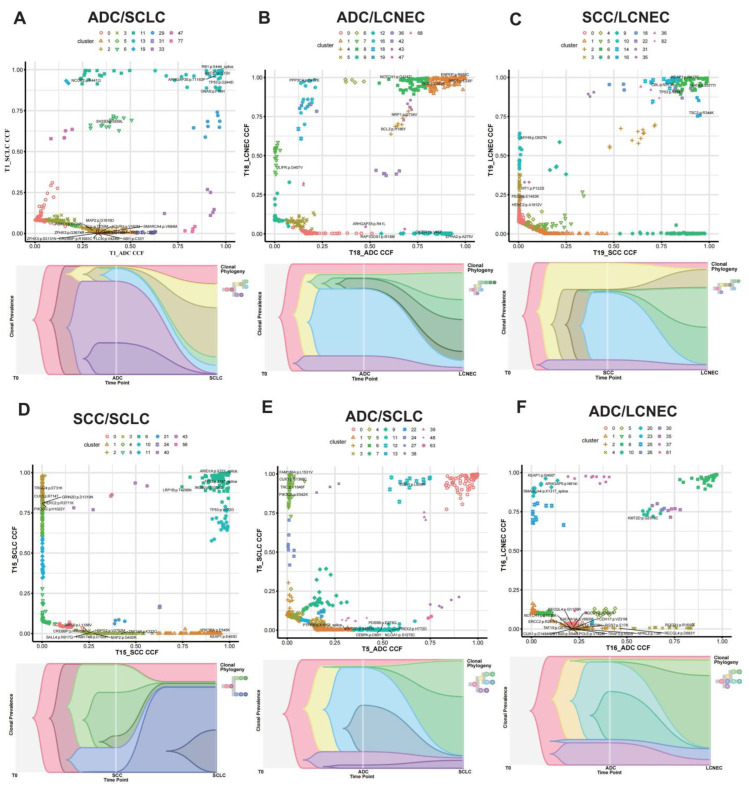
Representative clonal relationship between two tumor components of combined SCLC/ADC, LCNEC/ADC, LCNEC/SCC, and SCLC/SCC patients shown in a fish plot and a two-dimensional plot. (**A**–**D**) Representative patients who met criteria (i), in which possibly damaging clonal driver mutations were shared by two tumor components. (**E**) Representative patient who met criteria (ii), in which possibly damaging driver mutations were clonal in one tumor component but subclonal in another paired tumor component. (**F**) Representative patient who met criteria (iii), in which ≥2 clusters with passenger mutations were shared by two tumor components and were clonal in at least one tumor component. T0: default virtual point by software, CCF: cancer cell fraction.

**Figure 4:**
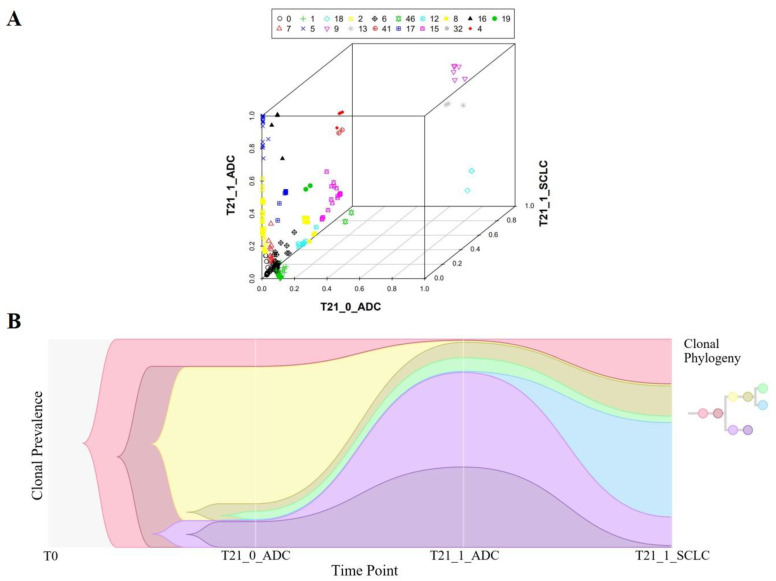
Clonal relationship between three tumor components of a combined SCLC/ADC patient shown in a three-dimensional plot and a fish plot. (**A**,**B**) Patient T21 who met criteria (iii), in which ≥2 clusters with passenger mutations were shared by three tumor components and were clonal in at least one tumor component.

Somatic mutations were used to investigate the clonal relationship between the paired tumor components of each patient. The distribution of clusters based on two-dimensional plotting of CCF is shown in Figure 3 and Figure 4 and Supplementary Figures S2 and S3. As shown, among the 15 SCLC/ADC patients, 12 patients met criteria (i), 2 patients met criteria (ii), and 1 patient met criteria (iii). Among the three LCNEC/ADC patients, two met criteria (i) and one patient met criteria (iii). Meanwhile, the two LCNEC/SCC and one SCLC/SCC patient met criteria (i). Furthermore, the fish plot of the clonal evolution analysis showed that the major clones of the two components in these combined SCLC/ADC, LCNEC/ADC, LCNEC/SCC, and SCLC/SCC patients were different (Figure 3 and Figure 4, Supplementary Figures S2 and S3). As shown, one component did not originate from the other paired component. Moreover, the clonal divergence of both components occurred early in tumorigenesis.

## 4. Discussion

In our study, *TP53*, *RB1*, and *EGFR* were the most common molecular alterations in C-NECs, and the mutation frequency was consistent with pure NECs except for *EGFR*. The frequency of *TP53* mutations assessed using WES was a little higher than that assessed for *p53* mutations using IHC. The concordance rate between IHC and WES for p53 and *TP53* was 81.0%, indicating that p53 expression in the IHC assay may reasonably correlate with the mutational data in previous studies [13] and in our study. For RB1, an alteration in protein expression was assessed using the IHC assay and a simultaneous absence of mutation was indicated by WES in our study, consistent with the results of other studies. However, the reason for this remained unclear. Derks et al. [14] generated homozygous gene deletions and measured gene expression using fluorescence in situ hybridization, but they could not explain the loss of expression in their wild-type samples. The inconsistent results may be attributed to frequent disruptions caused by genomic rearrangements in *RB1* and its breakpoints being located frequently in introns. Additionally, the evaluation of RB1 mutations using IHC was higher than using WES, and the IHC and WES analysis for Rb/*RB1* in each individual yielded a consistency rate of 61.9%. Therefore, when conditions permit, IHC and molecular detection must be used simultaneously; otherwise, IHC is better for detecting Rb.

Histologic transformation is regarded as the resistance mechanism against EGFR [28], ALK [29], or immunotherapy [18], and the frequency of transformation to SCLC is reported to be in the range of 3–14% [30,31]. Considering that the original *EGFR* mutation persisted after transformation, the primary ADC and the subsequent histology may have the same origin. In addition, Lin et al. [32] performed a WES analysis on four patients with C-SCLC and four patients with ADC transformed from small-cell carcinoma. The results showed that the transformed cases had a high consistency in *EGFR/TP53/RB1* mutations, suggesting that ADC and SCLC had the same histological origin. Similarly, regarding the combined carcinoma, researchers have reported that C-NEC has common EGFR mutations in both NEC and non-NEC components, suggesting that these two components may originate from cells of the same origin [33]. In addition, previous work has also identified that the two components of combined carcinoma are from a common cellular origin [32,34]. In our study, the clonal analysis and high consistency in *EGFR/TP53/RB1* mutation frequencies support the hypothesis that the two components originated from the same clone. Despite the C-NEC cases enrolled in our study being unable to reflect the sequence of lesion development, our study and previous studies [32,33,35] reached the same conclusion, that the two different components in C-NEC or transformed cases share a common histogenetic origin.

Therefore, based on the high frequency of *EGFR* in the post-transformed cases (almost SCLC) [15,30,36,37], the abnormal Rb expression in the non-NEC (mainly ADC) [32], and the above discussions about the reported transformation cases (Table S2) [15,17,18,22,32,36,38,39], the C-NECs all had a common cellular origin. From the perspective of pathologists, we speculate that the two hypotheses regarding HT may be identical, with different understandings between pathologists and clinicians. Thus, it may be reasonable for patients with non-frequent SCLC features (never smoke, *EGFR* mutations) to receive secondary biopsies or to undergo surgical resection to detect possible potential combined SCLC/NSCLC. Misdiagnosis is also possible when the histological sample is taken partially, as case T21 was initially diagnosed as SCLC. Therefore, assessing the status of the *p53/Rb* and *EGFR* genes in such cases would help uncover any potential combined component. In addition, *EGFR/TP53/RB1*-mutant lung cancers are also at unique risk of HT [40], indicating that assessing the statuses of *p53/Rb*/*EGFR* genes is crucial.

Apart from its clinical value, we proposed the relationship between the so-called “SCLC transformation” in clinical and C-NEC because a subtype classification for SCLC has recently been proposed based on the high levels of key transcriptional regulators, namely *ASCL1* (SCLC-A), *NEUROD1* (SCLC-N), *POU2F3* (SCLC-P), and *YAP1* (SCLC-Y) [41]. Irlend et al. observed that MYC activates NOTCH signaling to dedifferentiate tumor cells from ASCL1+ to NEUROD1+ to the YAP1+ state, and they proposed that SCLC molecular subtypes are not distinct but instead show a dynamic stage of tumor evolution [42]. Doron Tolomeo et al. [43] found that plasmacytoma variant translocation 1 (PVT1) transcripts underlie a functional connection between MYC and YAP1/POU2F3, suggesting that they contribute to the transcriptional landscape associated with MYC amplification. Therefore, we speculate that SCLC transformation may be more suitable for the transformation of the four molecular subtypes. Better understanding the role and implications of such genes may provide a comprehensive molecular view of SCLC and its transformations. Although our team has already published relevant discoveries on the molecular subtype of SCLC [44], given the small sample size of this study, collaboration with multiple centers and laboratories to further verify these data is warranted in the future.

Though our study illustrates the correlation between C-NEC and the so-called HT, this study also has a few limitations. First, this study involved a relatively small number of patients. The small sample size may have resulted in selection bias. In addition, the number of samples with preoperative biopsy data in combined cases was small due to the lack of residual tissue for further WES detection. Further study on the correlation is warranted.

## 5. Conclusions

The molecular analysis of C-NEC cases in this study and the HT cases reported in the literature reveals that these cases show a high degree of consistency for a common histogenetic origin, which may reflect different understandings of the same tumor between pathologists and clinicians. Although IHC is relatively more sensitive in detecting the status of the *RB1* gene, WES is more accurate for detecting the status of the *TP53* gene. Detection of *p53/Rb* mutations and evaluation of the status of the *EGFR* gene would help identify potential transformed cases or cases with potentially combined components as early as possible.

## Figures and Tables

**Table 1:** Clinicopathologic characteristics of 21 C-NECs.

Variable	N (%)	C-SCLC		C-LCNEC	
		SCLC/ADC (n = 15)	SCLC/SCC (n = 1)	LCNEC/ADC (n = 3)	LCNEC/SCC (n = 2)
Gender					
Male	18 (85.7)	13		5	
Female	3 (14.3)	3		0	
Age (median)					
≤62y	11 (52.4)	10		1	
>62y	10 (47.6)	6		4	
Mitotic Figure (NE)					
≤31	11 (52.4)	9		2	
>31	10 (47.6)	7		3	
Mitotic Figure (non-NE)					
≤2	14 (66.7)	11		3	
>2	7 (33.3)	5		2	
Tumor size (cm)					
≤3.4	10 (47.6)	8		2	
>3.4	11 (52.4)	8		3	
Tumor location					
Peripheral	10 (47.6)	9		1	
Central	11 (52.4)	7		4	
Necrosis					
Presence	12 (57.1)	7		5	
Absence	9 (42.9)	9		0	
Pleural invasion					
Yes	12 (57.1)	9		3	
No	9 (42.9)	7		2	
Vascular invasion					
Yes	8 (38.1)	6		2	
No	13 (61.9)	10		3	
Nerve invasion					
Yes	0 (0.0)	0		0	
No	21 (100.0)	16		5	
STAS					
Yes	2 (9.5)	2		0	
No	19 (90.5)	14		5	
Neoadjuvant therapy					
Yes	6 (28.6)	4		2	
No	15 (71.4)	12		3	
Smoke					
Yes	1 (4.8)	1		0	
Pre-smoking					
Within 1 year	3 (14.3)	2		1	
Within 1–3 years	3 (14.3)	1		2	
Above 3 years	4 (19.0)	3		1	
Never	10 (47.6)	9		1	
Family history					
Yes	5 (23.8)	3		2	
No	16 (76.2)	13		3	
T stage					
T1a	1 (4.8)	1		0	
T1b	1 (4.8)	1		0	
T1c	7 (33.3)	6		1	
T2a	8 (38.1)	5		3	
T2b	1 (4.8)	1		0	
T3	2 (9.4)	1		1	
T4	1 (4.8)	1		0	
n					
0	15 (71.4)	12		3	
N1a	1 (4.8)	0		1	
N1b	2 (9.4)	2		0	
N2a1	1 (4.8)	0		1	
N2a2	1 (4.8)	1		0	
N2b	1 (4.8)	1		0	
Clinical stage					
IA1	0 (0.0)	0		0	
IA2	1 (4.8)	1		0	
IA3	3 (14.3)	3		0	
IB	7 (33.3)	5		2	
IIA	1 (4.8)	1		0	
IIB	5 (23.8)	3		2	
IIIA	3 (14.3)	2		1	
IV	1 (4.8)	1		0	

**Table 2:** The expression of p53 and Rb assessed using IHC in different components of each case.

	Rb IHC Expression				
p53 IHC expression		Inconsistent expression	Consistent mutant expression	Consistent wild-type expression	Total
	Inconsistent expression	1	2	1	4
	Consistent mutant expression	2	9	3	14
	Consistent wild-type expression	0	1	2	3
Total		3	12	6	21

**Table 3:** The status of *TP53/RB1* assessed using WES in two different components of each case.

	*RB1* Status				
*TP53* status		Inconsistent status	Consistent mutant type	Consistent wild type	Total
	Inconsistent status	1	0	1	2
	Consistent mutant type	3	3	10	16
	Consistent wild type	2	0	1	3
Total		6	3	12	21

**Table 4:** The concordance rate between IHC and WES for *TP53*/p53 and *RB1*/Rb.

Category	Combined NECs (%)		Concordance Rate (%)
	C-SCLC	C-LCNEC	
*TP53*	18/21 (85.7)		17/21 (81.0)
	14/16 (87.5)	4/5 (80.0)	
p53 IHC	18/21 (85.7)		
	14/16 (87.5)	4/5 (80.0)	
*RB1*	9/21 (42.9)		13/21 (61.9)
	7/16 (43.8)	2/5 (40.0)	
Rb IHC	15/21 (71.4)		
	13/16 (81.3)	2/5 (40.0)	
*EGFR*	9/21 (42.9)		/
	8/16 (50.0)	1/5 (20.0)	

## Data Availability

The raw data are available upon request to the following e-mail address: hyuewang@163.com.

## Supplementary Materials

The following supporting information can be downloaded at: https://www.mdpi.com/article/10.3390/cancers15235649/s1, Figure S1. A. Case T21, which showed TP53 in frame del mutation in three samples of C-SCLC. EGFR was detected mutation in SCLC component of C-SCLC, no mutation was observed in ADC component. B. The common molecular alterations for combined LCNEC/ADC, combined LCNEC/SCC, and combined SCLC/SCC. Figure S2. The clonal relationship between 2 tumor components of these combined SCLC/ADC patients shown by fish plot and the two-dimensional plot. (A-K) Patients who met criteria i, in which possibly damaging clonal driver mutations were shared by two tumor components. (L) The patient who met criteria ii, in which possibly damaging driver mutations were clonal in one tumor component but subclonal in another paired tumor component. Figure S3. The clonal relationship between 2 tumor components of combined LCNEC/ADC and LCNEC/SCC patients shown by fish plot and the two-dimensional plot. (A,B) Patients who met criteria i, in which possibly damaging clonal driver mutations were shared by two tumor components; Table S1: The detailed information for the 21 C-NECs. Table S2: Reported cases of transformation from non-NEC to NEC.
