# Supplementary material for: Clonality Analysis for the Relationship between the Pulmonary Combined Neuroendocrine Carcinoma and “the So-Called Reported Histologic Transformation”
Source: Cancers (Basel). 2023 Nov 29;15(23):5649. doi: 10.3390/cancers15235649 (PMC10705092; doi:10.3390/cancers15235649)
Supplement: Supplementary file 1 [file cancers-15-05649-s001.zip › cancers-2696729-supplementary.pdf]

Table S1. The detailed information for the 21 C-NECs.

Sample	Age	Gender	Smoke	TNM	Histologic type	Proportion (%)	Preoperative biopsy (Yes, diagnosis/No)	ARMS PCR	Mutation analysis (WES)						IHC	
								EGFR	EGFR	TP53	RB1	NOTCH1	PIK3CA/PTEN/AKT1/TSC1	Others	p53	Rb
T1	75	M	Never	T3N0	SCLC;	90	Yes, ADC	Unknown	None	p.G244D&p.R175H	p.X444_splice	None	None	TERT p.S1055L	95%, diffuse+ (1)	50%, Moderate+ (0)
					ADC (lepidic)	10		Unknown	None	p.G244D&p.R175H	p.X444_splice	None	None	None	10%, moderate+ (0)	100%, - (1)
T2	61	M	ex-smoker > 3 year	T1cN0	SCLC	20	No	Unknown	p.G719A	p.Y220C&p.D207A	None	None	None	None	90%, diffuse+ (1)	100%, - (1)
					ADC (lepidic)	80		Unknown	p.G719A	None	p.X463_splice	None	None	None	25%, moderate+ (0)	100%, - (1)
T3	83	F	Never	T1cN0	SCLC	70	No	18 exon (G719A) & 21(L861Q)	p.G719A&p.L861Q	p.P191T& p.P177L	None	None	PTEN p.H196N	TERT p.V640M	90%, diffuse+ (1)	100%, - (1)
					ADC (micropapillary)	30		18 exon (G719A) & 21(L861Q)	p.G719A&p.L861Q	p.P177L	p.X463_splice	NOTCH1 p.S2136L	None	None	90%, diffuse+ (1)	75%, Weak+ (0)
T4	68	F	Never	T2aN0	SCLC	5	No	L858R	p.L858R	None	p.Q736*	None	PIK3CA p.E109K & p.E453Q	None	10%, Weak+ (0)	100%, - (1)
					ADC (acinar, lepidic)	95 (65, 35)		L858R	p.L858R	None	None	None	None	None	25%, diffuse+ (0)	100%, - (1)
T5	62	M	smoker	T1cN1b	SCLC	30	No	L858R	p.L858R	p.R273C	None	None	PIK3CA p.E542K	None	95%, diffuse+ (1)	100%, - (1)
					ADC (lepidic)	70		L858R	p.L858R	p.R273C	None	None	None	TERT p.S656L	95%, diffuse+ (1)	100%, - (1)
T6	60	M	Never	T1cN1b	SCLC	10	Yes, ADC	L858R	p.L858R	p.V73Wfs*50	p.D338E	None	None	None	100%, - (1)	100%, - (1)
					ADC (lepidic, acinar, micropapillary, papil-	90 (65,25, 8,2)		L858R	p.L858R	p.V73Wfs*50	None	None	None	None	100%, - (1)	100%, - (1)

					lary)											
T7	76	M	Never	T2aN0	SCLC	90	No	Un-known	p.E746_A750del	None	None	None	PTEN p.T319Nfs*4	MYCL p.D83N	100%, - (1)	100%, - (1)
					ADC (acinar)	10		Un-known	None	None	None	NOTCH1 p.R234C	PTEN p.T319Nfs*4	None	100%, - (1)	100%, - (1)
T8	61	M	ex-smoker = 3 years	T2aN0	SCLC	95	No	Un-known	None	p.R158G	None	None	AKT1 p.E247K	None	95%, diffuse+ (1)	90%, Weak+ (0)
					ADC (lepidic)	5		Un-known	None	p.R158G	None	None	None	None	95%, diffuse+ (1)	90%, Weak+ (0)
T9	72	F	Never	T1cN2a2	SCLC	25	Liver puncture, SCLC metasta-sis	19 del	None	p.R175H	None	None	None	None	20%, moder-ate+ (0)	100%, - (1)
					ADC (lepidic, acinar)	75 (80, 20)		19 del	p.L747_P753delinsS	p.R175H	None	None	None	TERT p.G861R	95%, diffuse+ (1)	100%, - (1)
T10	59	M	Never	T1aN2b	SCLC	45	Yes, no tumor cell	19 del	None	p.H193Y	None	NOTCH1 p.G1034D	None	None	95%, diffuse+ (1)	100%, - (1)
					ADC	55		19 del	None	p.H193Y	None	None	TSC1 p.P323L	None	95%, diffuse+ (1)	100%, - (1)
T11	56	M	Never	T2bN0	SCLC	60	No	No	None	p.Y205C	None	None	None	None	60%, weak+ (0)	30%, weak+ (0)
					ADC (aci-nar)	40		No	None	p.Y205C	None	None	None	None	90%, diffuse+ (1)	30%, weak+ (0)
T12	60	M	ex-smok-er > 3 year	T1bN0	SCLC	90	No	No	None	p.F54Sfs*69	p.G449R	None	None	None	100%, - (1)	100%, - (1)
					ADC (aci-nar)	10		No	None	p.F54Sfs*69	p.G449R	None	None	None	100%, - (1)	100%, - (1)
T13	54	M	ex-smok-er > 3 year	T1cN0	SCLC	30	No	No	None	p.Q375*	p.Q257*	None	None	None	100%, - (1)	100%, - (1)
					ADC (papillary, acinar, lepidic)	70 (70,20, 10)		No	None	p.Q375*	p.Q257*	None	None	None	100%, - (1)	100%, - (1)
T14	55	M	ex-smoker = 0.3 year	T2aN0	SCLC	85	Yes, Large cell carcino-ma	Un-known	None	p.V274D	None	None	None	None	95%, diffuse+ (1)	100%, - (1)
					ADC	15		Un-known	None	p.V274D	None	None	None	None	95%, diffuse+ (1)	100%, - (1)
T15	68	M	ex-smoker = 0.2 year	T2aN0	SCLC	80	Yes, SCC	Un-known	None	p.X187_splice&p.V172D	None	None	None	None	20%, moder-ate+ (0)	70%, moder-ate+ (0)
					SCC	20		Un-known	None	p.X187_splice&p.V172D	None	None	PIK3CA p.E545K	None	40%, moder-ate+ (0)	70%, moder-ate+ (0)
T16	73	M	ex-smok-	T3N0	LCNEC	95	No	No	None	None	None	NOTCH1 p.A1471V	None	None	60%, moder-ate+ (0)	70%, moder-ate+ (0)

			er > 3 years		ADC (acinar)	5		No	None	*p.R267W*	None	*NOTCH1* p.A1471V	None	*MYCN p.A174T*	60%, moderate+ (0)	50%, moderate+ (0)
T17	59	M	ex-smoker = 0.1 year	T2aN0	LCNEC	95	Yes, ADC	No	None	*p.V274F*	None	*NOTCH2 p.G1612V*	None	None	95%, diffuse+ (1)	100%, - (1)
					ADC	5		No	*p.D1168H*	*p.V274F*	None	*NOTCH2 p.G1612V& p.H1517R*	None	None	95%, diffuse+ (1)	100%, - (1)
T18	68	M	ex-smoker = 1 year	T2aN0	LCNEC	95	Yes, ADC	No	None	None	None	*NOTCH1* p.Q1247*	None	None	100%- (1)	30%, weak+ (0)
					ADC (acinar)	5		No	None	None	*p.K8N*	*NOTCH1* p.Q1247*	None	None	100%- (1)	30%, weak+ (0)
T19	74	M	Never	T2aN1a	LCNEC	70	Yes, LCNEC	Unknown	None	*p.Y234C*	*p.V654Sfs*14*	None	None	None	95%, diffuse+ (1)	100%, - (1)
					SCC	30		Unknown	None	*p.Y234C*	None	None	None	None	95%, diffuse+ (1)	30%, moderate+ (0)
T20	67	M	ex-smoker = 1 year	T1cN2a1	LCNEC	45	No	Unknown	None	*p.C242Y*	None	None	None	None	95%, diffuse+ (1)	70%, weak+ (0)
					SCC	55		Unknown	None	*p.C242Y*	None	None	None	None	95%, diffuse+ (1)	70%, weak+ (0)
T21	35	M	Never	T4N0M1	SCLC	98	Yes, ADC	*19 del*	*p.E746_A750del*	*p.R337_M340del*	None	None	*PIK3CA p.N345K*	None	95%, diffuse+ (1)	100%, - (1)
					ADC	2		*19 del*	None	*p.R337_M340del*	None	None	None	None	95%, diffuse+ (1)	100%, - (1)

IHC, “1” represent mutant type; “0” represent “wild type”.

Table S2. Reported cases of transformation from non-NEC to NEC.

Case, year	Age	Gender	smoking	Sample type, before	Sample type, after	Diagnostic samples at first (before therapy)			Transformation samples (after therapy)		
						TP53/p53	RB1/Rb	EGFR/ALK	TP53/p53	RB1/Rb	EGFR/ALK
Zakowski MF [15], 2006	45y	F	never	Biopsy, bronchial washes	biopsy	/	/	/	/	/	18-bp deletion in exon 19
Morinaga R [38], 2007	46	F	never	Biopsy,	Biopsy,	/	/	/	/	/	15-bp deletion in exon 19

van Riel S [36], 2012	42	F	never	cytology	Biopsy,	/	/	/			exon 19 deletion
Popat S [39], 2013	46	F	never	lobectomy	Biopsy	/	/	exon 19 deletion	/	/	*EGFR* exon 19 deletion
Fujita S [17], 2016	67	F	never	biopsy	biopsy	/	/	ALK rearrangement	/	/	ALK rearrangement
Abdallah N [18], 2018											
Case 1	65	M	35y	fluid cytology	biopsy	/	/	*EGFR* wild-type; no *ALK* translocation	/	/	/
Case 2	68	M	NA	wedge resection	biopsy	/	/	/	/	/	/
Mong-Wei Lin [32], 2018											
Case 5	64	M	Never	computed tomography-guided biopsy	surgical resection	wild	mutation	Exon 19 del (p.E746_A750del)	Wild	mutation	Exon 19 del (p.E746_A750del)
Case 6	66	F	Never	computed tomography-guided biopsy	surgical resection	mutation	mutation	p.L858R	mutation	mutation	p.L858R
Case 7	57	F	Never	surgical biopsy	surgical resection	mutation	Mutation	p.L858R	Mutation	Mutation	p.L858R
Case 8	63	F	never	surgical resection	surgical resection	mutation	mutation	Exon 19 del (p.E746_A750del)	mutation	mutation	Exon 19 del (p.E746_A750del)
Xie T [22], 2020											
P2	70	F	never	Biopsy	Biopsy	mutation	wild	Ex19Del	mutation	wild	Ex19Del
P4	33	F	never	Biopsy	Biopsy	mutation	mutation	Ex18G719X	mutation	mutation	Ex18G719X
P5	73	M	never	Biopsy	Biopsy	mutation	wild	Ex19Del	mutation	wild	Ex19Del

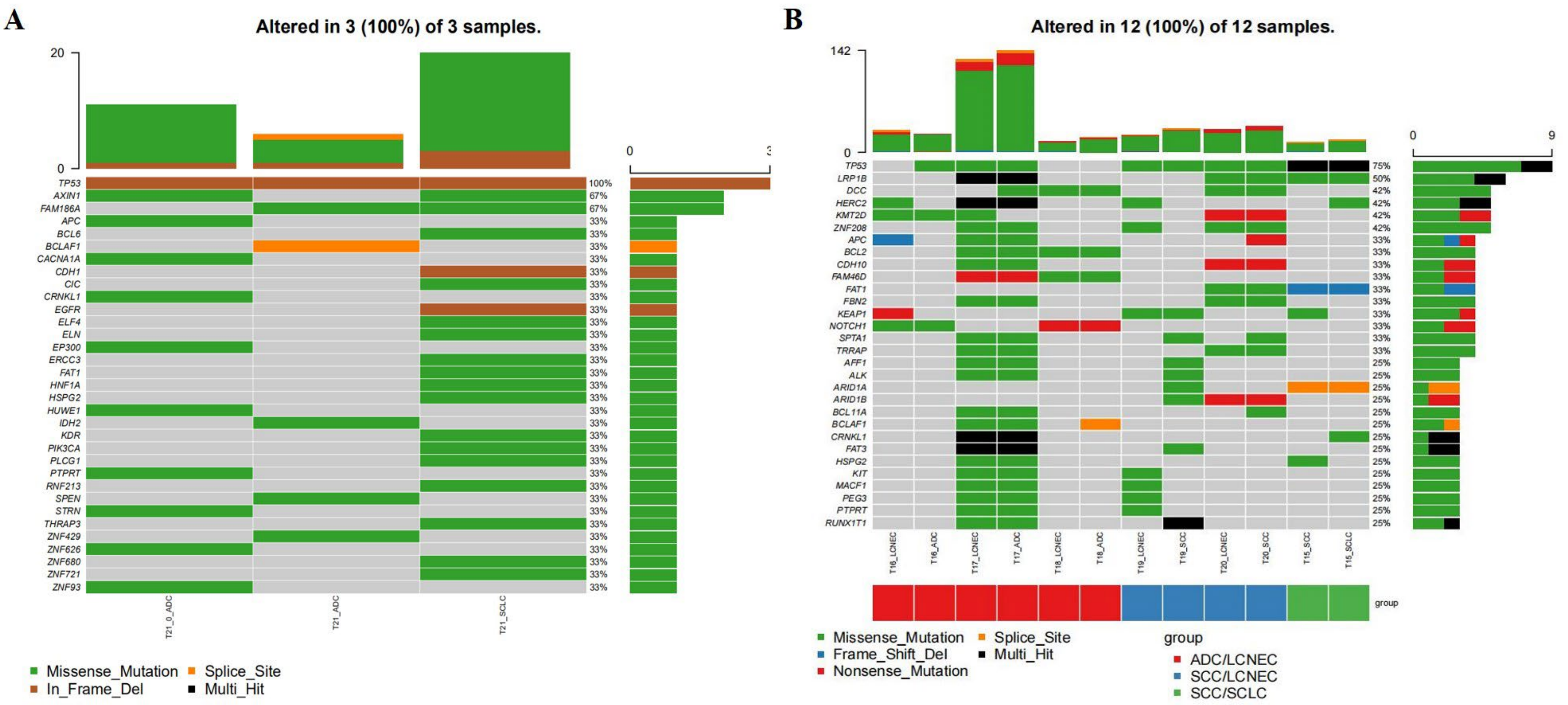

Supplementary Figure S1. A. Case T21, which showed *TP53* in frame del mutation in three samples of C-SCLC. *EGFR* was detected mutation in SCLC component of C-SCLC, no mutation was observed in ADC component. **B.** The common molecular alterations for combined LCNEC/ADC, combined LCNEC/SCC, and combined SCLC/SCC.

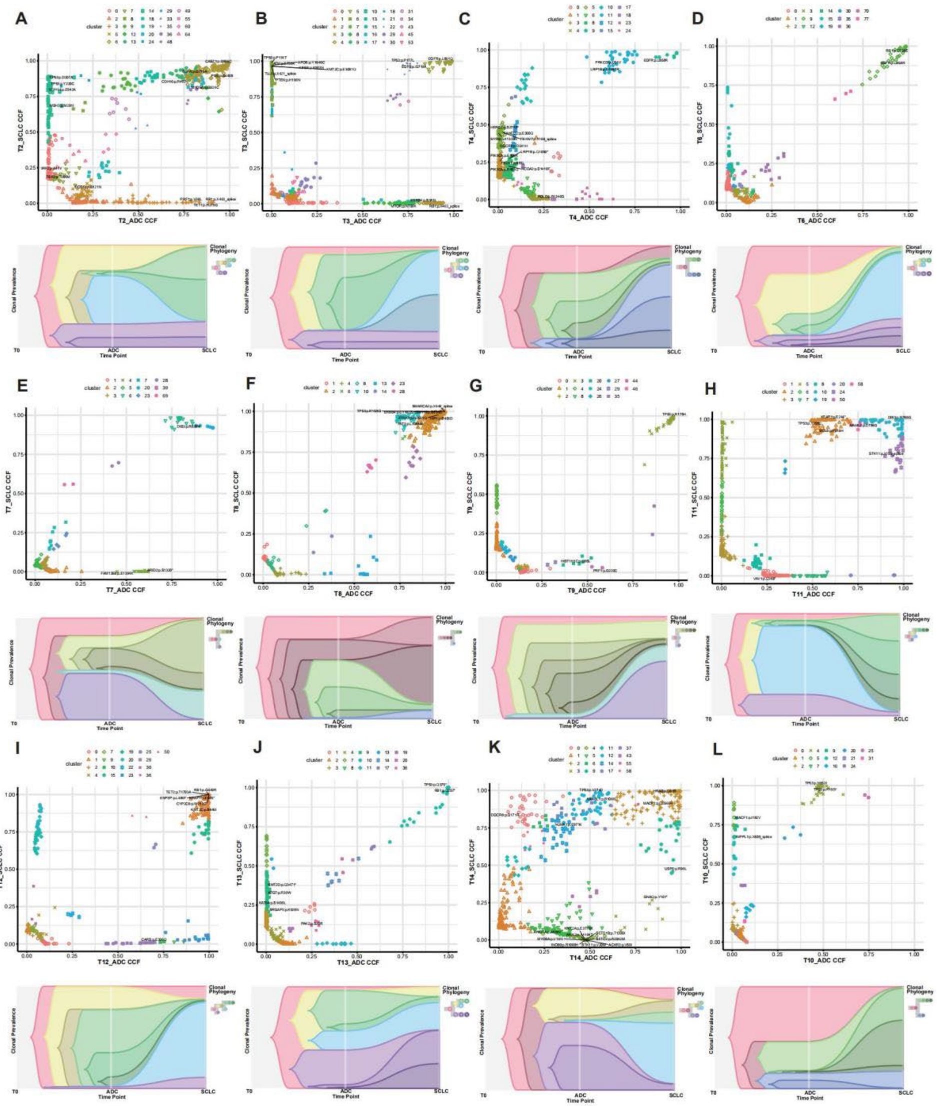

Supplementary Figure S2. The clonal relationship between 2 tumor components of these combined SCLC/ADC patients shown by fish plot and the two-dimensional plot. (**A-K)** Patients who met criteria i, in which possibly damaging clonal driver mutations were shared by two tumor components. **(L)** The patient who met criteria ii, in which possibly damaging driver mutations were clonal in one tumor component but subclonal in another paired tumor component.

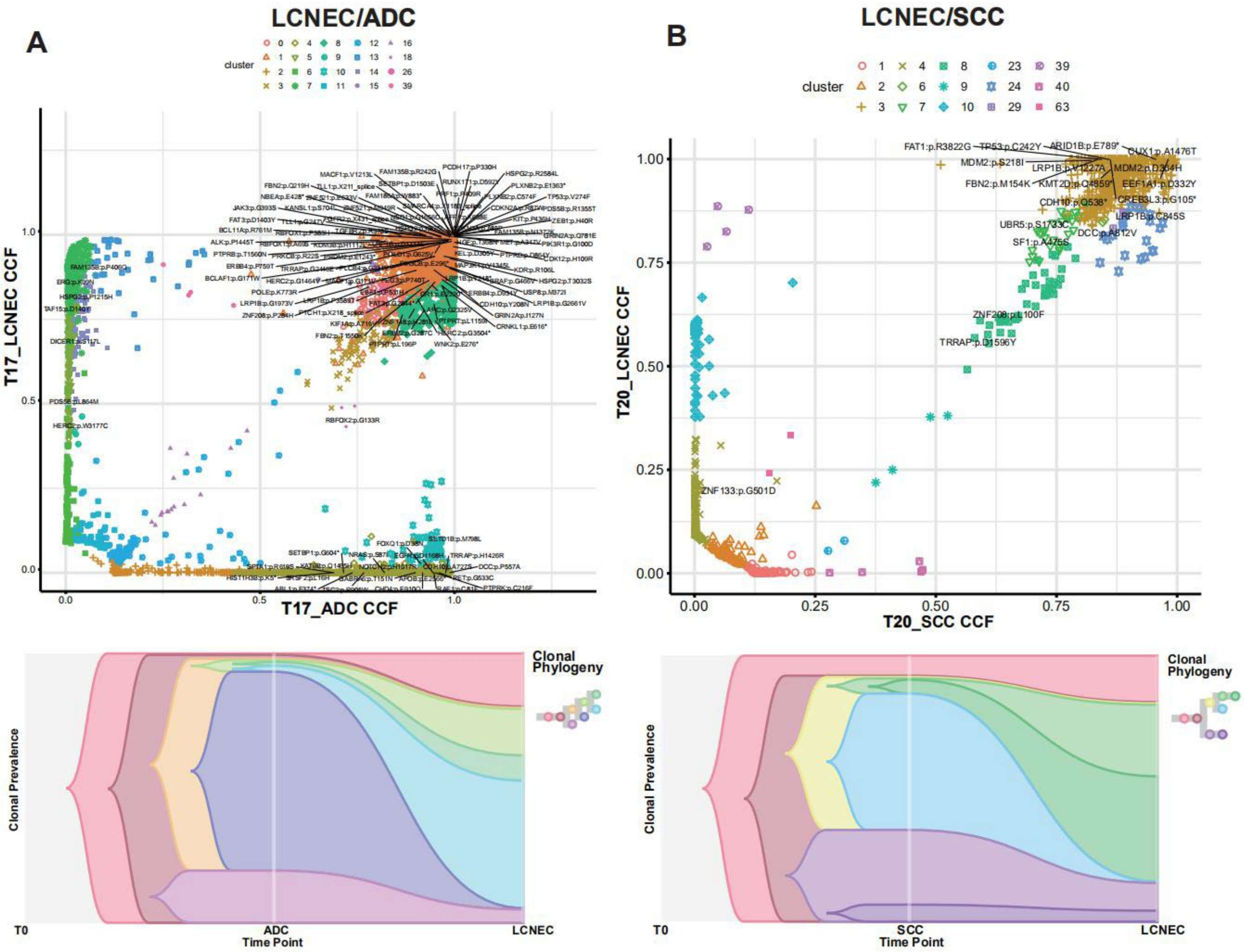

Supplementary Figure S3. The clonal relationship between 2 tumor components of combined LCNEC/ADC and LCNEC/SCC patients shown by fish plot and the two-dimensional plot. **(A-B)** Patients who met criteria i, in which possibly damaging clonal driver mutations were shared by two tumor components.
